# High Dose of Metformin Decreases Susceptibility to Occlusive Arterial Thrombosis in Diabetic Mice

**Published:** 2023-10-16

**Authors:** Roberto I. Mota Alvidrez, Gowtham K. Annarapu, Amudan J. Srinivasan, Zeyu Liu, Hamza O. Yazdani, Deidre Nolfi-Donegan, Richard L. Simmons, Sruti Shiva, Matthew D. Neal

**Affiliations:** 1 Trauma and Transfusion Medicine Research Center, Department of Surgery, University of Pittsburgh Medical Center, Pittsburgh, Pennsylvania, USA 15213; 2 McGowan Institute for Regenerative Medicine, University of Pittsburgh, Pittsburgh, Pennsylvania, USA 15213; 3 Vascular Medicine Institute, University of Pittsburgh, Pittsburgh, Pennsylvania, USA 15213; 4 Department of Pathology, University of Pittsburgh, Pittsburgh, Pennsylvania, USA 15213; 5 Pittsburgh Liver Research Center, University of Pittsburgh, Pittsburgh, Pennsylvania, USA 15213; 6 Department of Biology, University of Pittsburgh, Pittsburgh, Pennsylvania, USA 15213; 7 Department of Pharmacology & Chemical Biology, University of Pittsburgh, Pittsburgh, Pennsylvania, USA 15213; 8 Clinical and Translational Science Center, University of New Mexico, USA 87131; 9 Department of Pharmaceutical Sciences, University of New Mexico, USA 87131

**Keywords:** Metformin, arterial thrombosis, Diabetes, platelets, ADP

## Abstract

**Introduction:**

Metformin is the most prescribed medication in Diabetes Mellitus(DM). Metformin has shown to decrease mean platelet volume, with promising antiplatelet effects. High doses of Metformin have also been associated with hypercoagulation. We hypothesize that Metformin will protect DM mice from occlusive arterial thrombus formation by altering platelet activation and mitochondrial bioenergetics.

**Methods:**

DM was developed by low dose of Streptozotocin, non-DM (healthy) mice are controls. Either vehicle or Metformin was administered twice daily via oral gavage for 7-days. Ferric chloride (FeCl3) arterial thrombosis and tail bleeding time were performed. Whole blood aggregometry, platelet activation/adhesion and mitochondrial bioenergetics were evaluated.

**Results:**

Metformin decreased susceptibility of DM mice to arterial thrombosis. Platelet bioenergetics show DM mice have increased platelet mitochondrial respiration, but no differences were observed with Metformin treatment. In non-DM (healthy) mice, Metformin modulated ADP-dependent increase in platelet adhesion. Non-DM (healthy) mice, Metformin shortens bleeding time with faster thrombotic occlusion. Metformin also increased platelet mitochondrial maximal respiration and spare respiratory capacity uniquely in non-DM (healthy) mice.

**Conclusion:**

Metformin regulates platelet bioenergetics and ADP-mediated platelet function in DM mice which attenuates susceptibility to arterial thrombosis. Future studies will evaluate clinically relevant doses of Metformin that regulates thrombotic function in diabetic platelets.

## 1. Introduction

Metformin is the most common first-line drug prescribed in DM patients [1]. AMPKa is the main activated pathway targeted by antidiabetic therapeutics [2]. AMPK is an established regulator of platelet function [3, 4]. Higher doses of Metformin are prescribed to DM due to poor glucose control [5, 6]. Same dose-dependent effect has been observed in murine models [7]. Platelets from diabetic patients have pro-thrombotic susceptibility with distinct response to different platelet stimulants [8]. High doses of Metformin (400mg/kg/day, this dose is routinely used in preclinical models) have shown to prevent arterial/venous thrombosis in diabetic rats by inhibiting extracellular mitochondrial DNA [9]. Metformin has shown to affect platelet aggregation velocity and adhesion via ADP [10]. Metformin has been uniquely linked with potential beneficial antiplatelet effects in gestational diabetes [11]. Women with Gestational Diabetes received Metformin or placebo around 29 weeks of gestation. Soluble P-selectin was increased in the Metformin group with only a slight increase in soluble P-selectin in both placebo and Metformin from baseline [12].

The dose and frequency of Metformin that confers efficacy and safety to be used in multiple disease models is still to be determined [6]. The use of standard doses of Metformin with combination therapies such as platelet inhibitors has not shown a synergistic benefit [13]. Contrasting evidence has shown that similar high doses of Metformin are linked to treatment-related toxicity [14, 15]. Contrasting findings are on high doses that alter coagulation cascade factors particularly by affecting liver function [16, 17].

Our study aims to test the effect of Metformin on the susceptibility to thrombosis in DM mice. The main hypothesis of our study is that high doses of Metformin will have a beneficial effect in decreasing susceptibility to occlusive thrombosis development in DM mice. Our findings illustrate the need for deeper studies of the beneficial antiplatelet dose- and time-dependent effects of Metformin in DM.

## 2. Materials and Methods

### 2.1 Low dose Streptozotocin (STZ) DM Mouse Model

C57/BL6 mice at 6 weeks of age were given 5-day low dose of STZ (572201-1GM, Millipore Sigma) Intraperitoneal (IP) 25mg/kg(18, 19) for DM mice model development. DM developed for 12 weeks. Non-DM (healthy) control mice were injected with saline IP for 5 days with the same amount of volume that Streptozotocin treated DM mice received. We performed routine mouse assessment as well as body weight and 16hr fasting glycemia (75840-798, VWR) and insulin quantification via ELISA (ab277390, Abcam) in all mice as a standard to corroborate the correct model development which is well described in the literature as a DM model.

### 2.2 In Vivo Studies

All mice were placed on a normal diet. Both DM and non-DM mice at 18 weeks of age, 12 weeks post Streptozotocin or saline IP injections, were randomized into 2 treatments with either Metformin or vehicle control. Metformin treatment was administered for 7 days using oral gavage (317240-5GM, Millipore Sigma) twice daily at a dose of 200mg/kg per dose. Autoclaved DI water (diluent, vehicle) was used as a treatment for controls using the same amount of volume that Metformin treated mice received. A cohort of mice were randomized after last day of treatment for whole blood sample collection from mice for ex vivo experiments. Blood was collected using collection tubes with 3.2% (0.109M) with sodium citrate in a 1:9 citrate:blood ratio (BD Biosciences).

### 2.3 Platelet Bioenergetics (Mitochondrial Respiration Assays)

After last day of Metformin or vehicle treatment, a cohort of DM and non-DM mice (N of 4-5 mice per group) were euthanized for platelet isolation from collected platelet-rich plasma. Platelet number was determined as described in Walkowiak et al.(20) Platelets (50 x 10^6^ per well) were loaded into an XF96 microplate with unbuffered Dulbecco’s Modified Eagle Medium (DMEM) and centrifuged at 1500g for 5 minutes. Platelets were treated with oligomycin A (2.5 μmol/L), carbonyl cyanide p-(trifluoro-methoxy) phenyl-hydrazone (FCCP; 0.7 μmol/L), and Rotenone (10 μmol/L). Oxygen consumption rate (OCR) was measured by XF analysis (21)(XF96, Seahorse Biosciences, Billerica, MA).

### 2.4 Flow cytometry analysis of platelet activation

After last day of Metformin or vehicle treatment, a cohort of DM and non-DM mice (N of 4-5 mice per group) were sacrificed for whole blood collection. Platelet activation was measured in mouse whole blood (10:40 ratio) stained with anti-CD41a-FITC, anti-CD62P (P-selectin)-APC and JON/A antibody-PE (GPIIb/IIIa). Quantification was performed using BD LSR Fortessa^™^ flow cytometer with BD FACSDiva^™^ Software and analyzed with Flowjo 10.0. Positive anti-mouse CD41a-FITC was identified as platelet population. Activated platelets were quantified as positive gated on CD41+ population.

### 2.5 FeCl3 injury and arterial thrombosis model

After last day of Metformin or vehicle treatment, a cohort of DM and non-DM mice (N of 3-5 mice per group) were anesthetized with 10mg/kg Etomidate/1.2g/kg Urethane. After aseptic surgical area preparation, incision from the level of the manubrium to the mandible was made. Left carotid artery was isolated for placement of a doppler flow probe (Transonic Systems Inc., Ithaca, NY). A small plastic frit was placed under the carotid artery just proximal to the flow probe and a baseline flow reading was taken prior to placement of filter paper (Whatman) soaked with a fresh solution of 6% iron (III) chloride for 3 minutes. Surgical field was washed three times with PBS. Occlusive thrombosis was defined as cessation of flow without resumption over a 2-minute period in a 30-minute timepoint (5 seconds of zero-amplitude observed).

### 2.6 Tail bleeding time model

After last day of Metformin or vehicle treatment, a cohort of DM and non-DM mice (N of 3-5 mice per group) were anesthetized and placed in a supine position under a heat pad. After aseptic technique and disinfecting, 1cm of the tail tip was cut with a scalpel using a straight motion. Tail was submerged in a conical tube with 50mL of prewarmed (37°C) 0.9% Saline solution. Total bleeding time (sec) was recorded until no rebleed occurred within 3 min [22].

### 2.7 Ex vivo whole blood impedance aggregometry

Whole blood collected from DM and non-DM mice (N of 3-5 mice per group) was diluted with saline (10:40 ratio) and treated with Metformin or vehicle control (DMSO) for 30 min at room temperature. Collagen at 1ug/mL of (Bio/Data^™^ 101562) or ADP (20uM) (Bio/Data^™^ 101312) were used as agonists. Data was collected using CHRONO-LOG^®^ Model 700 Aggregometer by measuring impedance and was analyzed using AGGRO/LINK^®^8 software.

### 2.8 Statistical Analysis

Results are presented as mean ± standard error. The number of biological samples and replicates are described in each assay. We have outlined the sample size for each assay in the methods outlined above. Three different cohorts of mice were used: FeCl3 injury model, Tail bleeding time assays and ex vivo whole blood assays. Each cohort was composed of a sample size of 3-6 per experiment, experiments were repeated 3 times. Due to some attrition in our model, the number of samples per group varied (each is described in the appropriate sections). One-way ANOVA with Tukey’s post hoc analysis was performed were appropriate, two-way ANOVA with Tukey’s post hoc analysis between treatment groups assessing mouse phenotype and treatment analysis of variance as well as ADP and Collagen stimulation in Metformin and Vehicle treatment analysis of variance particularly in Fig 2 were appropriate, two-tailed t-tests were also performed were appropriate using GraphPad for Mac (Version 9.0.1). Statistical significance was established at *: p<0.05, **: p=0.001-0.01, ***: p=0.0001-0.001, ****: p<0.0001.

## 3. Results

**Figure 1:**
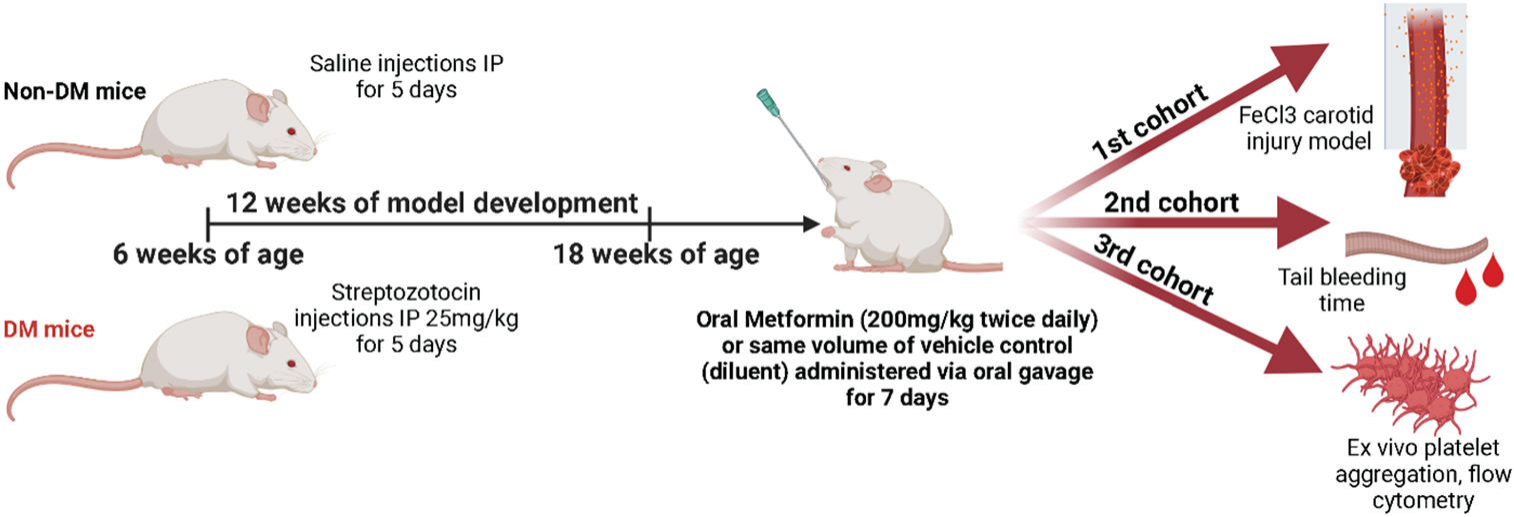
Diabetic mouse model development and oral Metformin studies methodologic scheme. Created with BioRender.

**Figure 2:**
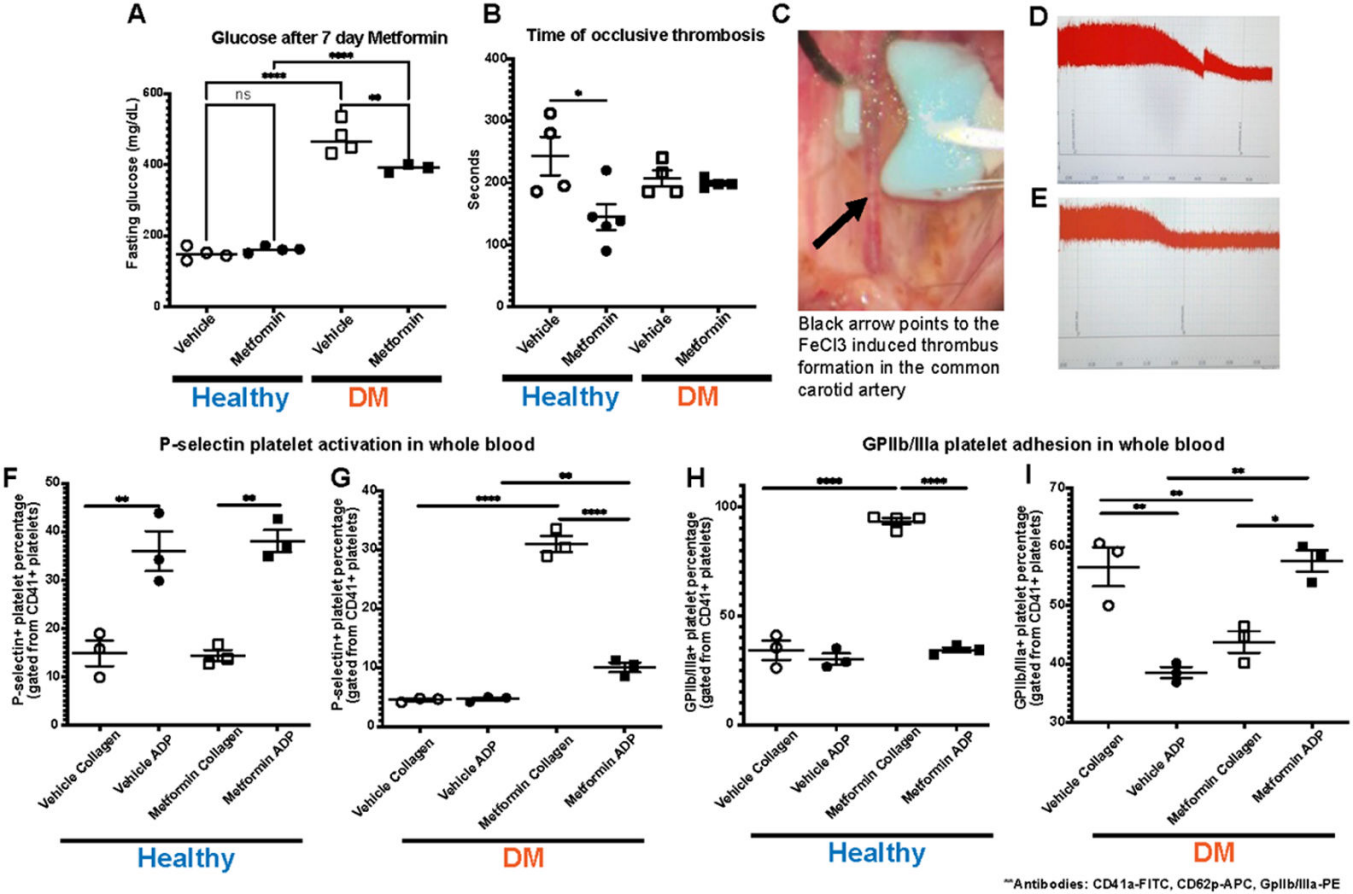
DM mice FeCl3 arterial thrombosis and flow cytometry analysis on 7-day oral Metformin (200mg/kg) treated mice. A) Effect of Metformin in vivo, decreased hyperglycemia in DM mice after 7 days with no effect in non-DM (healthy) mice(these mice did not exhibit hyperglycemia); N of 4-5 subjects per group was used for Two-way ANOVA: p 0.0258 with Tukey’s post hoc multiple comparisons was performed. B) Metformin shortens time to thrombus formation in normal mice while there is an apparent protective effect in DM mice; N of 4-5 subjects per group was used for Two-way ANOVA: p 0.0258 with Tukey’s post hoc multiple comparisons was performed. C) Representative image of FeCl3 surgical procedure with black arrow pointing to area of thrombus formation (white). D-E) Representative images of the readout of the time of occlusive thrombosis in the FeCl3 model in 7-day vehicle and Metformin treated non-DM mice. F-G) Metformin increased platelet activation via P-selectin expression mostly with Collagen stimulation in DM mice after Metformin treatment; N of 3-5 subjects per group was used for Two-way ANOVA: p <0.0001 with Tukey’s post hoc multiple comparisons was performed. H) In non-DM mice Metformin increases platelet adhesion (GpIIb/IIIa expression) under Collagen stimulation with a minimal effect under ADP simulation; N of 3 subjects per group was used for Two-way ANOVA: p 0.0005 with Tukey’s post hoc multiple comparisons was performed. I) In DM mice, Metformin decreased GpIIb/IIIa expression under Collagen stimulation with an inverse effect with ADP showing increase in GpIIb/IIIa expression; N of 3 subjects per group was used for Two-way ANOVA: p 0.0003 with Tukey’s post hoc multiple comparisons was performed.

In our studies, we treated established DM and non-DM mice with either Metformin or vehicle (control) for 7 days (Fig 1). High dose of Metformin decreased hyperglycemia only in DM mice despite the short treatment duration (Fig 2A). After 7-day treatment with Metformin, it was observed that DM mice did not have a difference in the time to FeCl3-induced occlusive thrombosis compared to vehicle treated mice (Fig 2B-2G). However, in non-DM (healthy) mice treated with Metformin, thrombosis occurred more rapidly with shorter time of occlusion (Fig 2C-E) compared to vehicle treated non-DM (healthy) mice (Fig 2B). In parallel, we performed flow cytometry analysis of markers of platelet activation and adhesion in both groups. We observed only a minimum non-statistically significant effect in platelet activation in non-DM (healthy) mice (Fig 2F) using flow cytometry. DM mice showed high platelet activation, more importantly to Collagen when treated with Metformin (Fig 2G). Baseline ADP platelet activation was lower in DM compared to non-DM (healthy) mice. Regarding platelet adhesion (GPIIb/IIIa expression), Metformin treatment in non-DM (healthy) mice increased GPIIb/IIIa with Collagen stimulation (Fig 2H). DM mice show that Metformin decreased GPIIb/IIIa under collagen stimulation but increased under ADP stimulation (Fig 2I).

**Figure 3:**
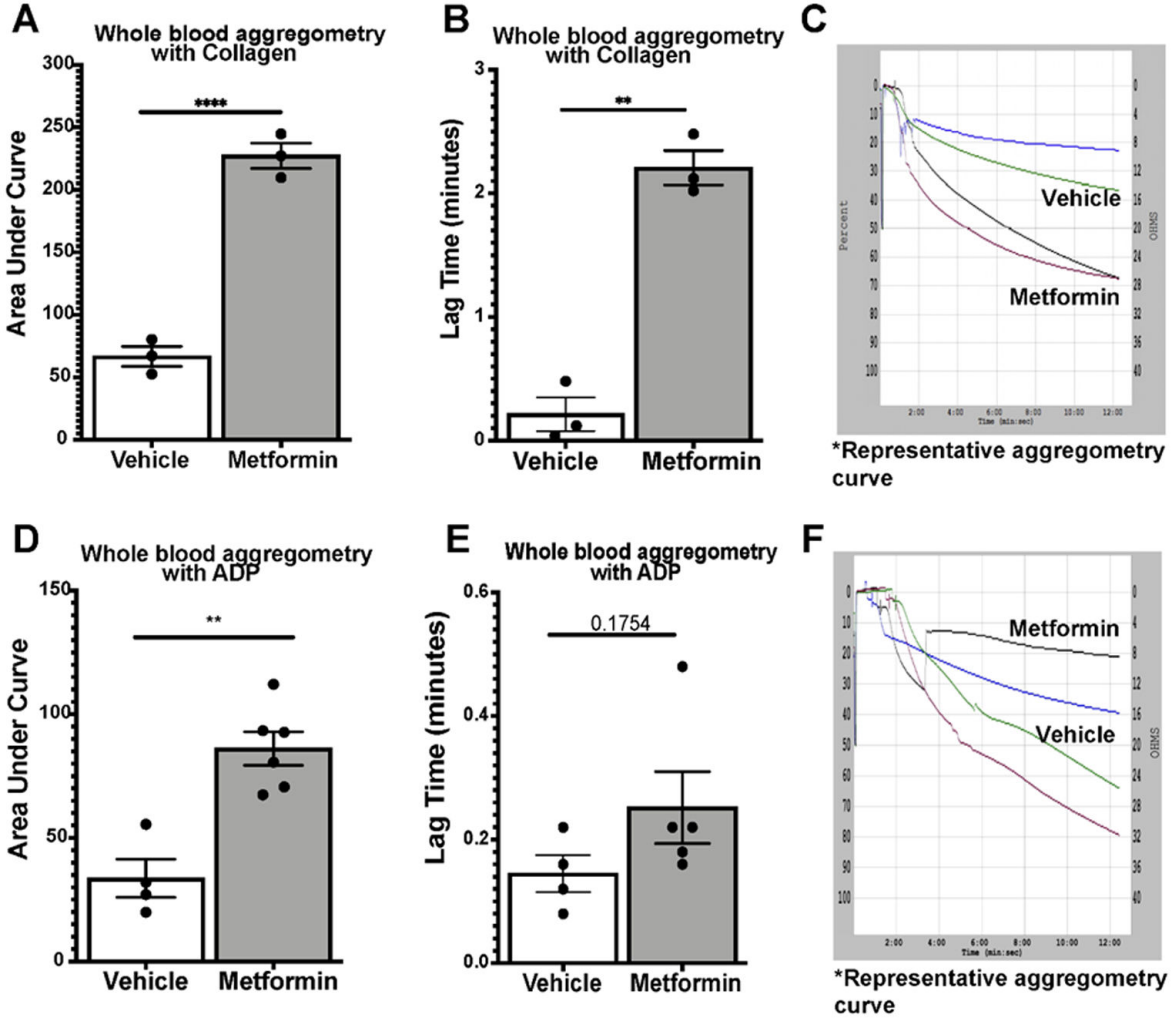
Whole blood 7-day oral Metformin (200mg/kg) platelet aggregometry with ADP/Ca+ and Collagen/Ca+ stimulation in non-DM (healthy) mice. A-C) Whole blood aggregometry with Collagen (1ug/mL). Data showed increased aggregation by AUC with Metformin treatment orally 200mg/kg twice daily compared to mice given vehicle for 7 days twice a day. Lag time is increased in Metformin treated mice; N of 3 subjects per group was used for Two-tailed t-test: p <0.0001 (A), p 0.0057 (B). D-F) Whole blood aggregometry with ADP (20uM) showed increased aggregation by AUC in mice given Metformin orally 200mg/kg twice daily compared to mice given vehicle for 7 days twice a day. Paradoxically to our earlier findings in increased lag time in Metformin treated mice stimulated with Collagen, when we used ADP as an agonist for aggregometry lag time increase effect was lost; N of 4-6 subjects per group was used for Two-tailed t-test: p <0.0001 (D), p 0.1754 (E).

**Figure 4:**
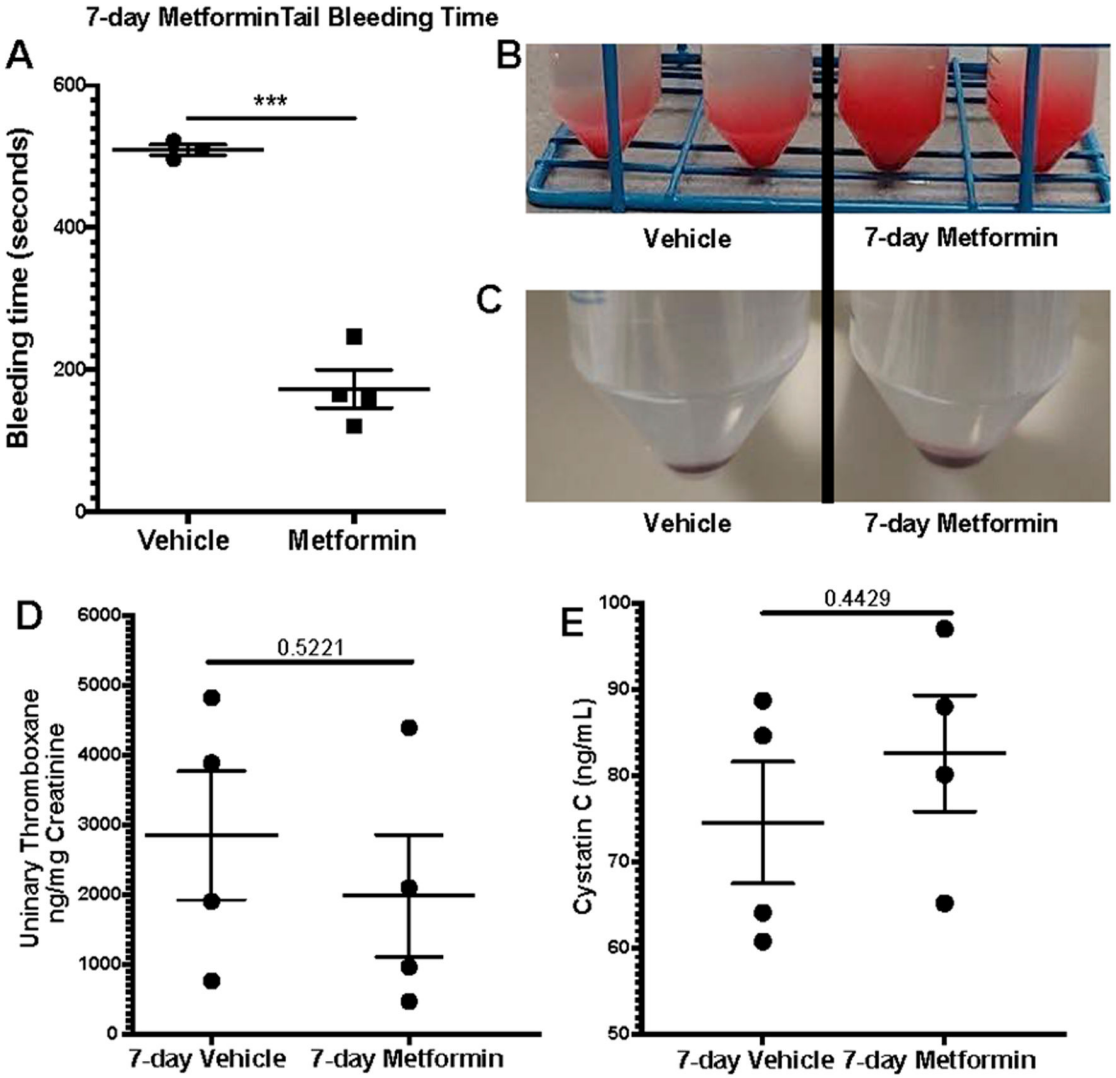
FeCl3 arterial thrombosis, bleeding time on 7-day Metformin non-DM treated mice. A) Oral Metformin treatment results in shorter tail bleeding time compared to vehicle treated mice; N of 3-4 subjects per group was used for Two-tailed t-test. B-C) Representative images show amount of blood collected during tail bleeding assays and sediment of cells after centrifugation. D-E) Platelet activation marker analysis with urinary Thromboxane and correlation with plasma Cystatin C levels for acute kidney injury development analysis showed no differences between vehicle and Metformin treated mice for 7 days; N of 4 subjects per group was used for Two-tailed t-test.

We assessed platelet aggregometry in non-DM (healthy) mice after 7-day treatment with Metformin compared to controls under both Collagen and ADP stimulation. There was a consistent increase in platelet aggregation (Fig 3A) and lag time (Fig 3B) in Metformin treated non-DM (healthy) mice regardless of length of stimulation (Fig 3C). Platelet aggregation using ADP, showed that Metformin also increased aggregation like Collagen (Fig 3D) but there was no difference in lag time (Fig 3E, 3F). As shown together with DM mice in Fig 2, non-DM (healthy) mice exhibited shorter time of occlusive thrombosis when treated with Metformin (Fig 2B-4E). We observed non-DM (healthy) mice have a shorter bleeding time when treated with Metformin compared to controls (Fig 4A). However, non-DM (healthy) mice showed higher total blood/cells loss during that short time (Fig 4B-C). Non-DM (healthy) mice had no development of kidney damage (Cystatin C) or associated changes in platelet activation evidenced by urinary thromboxane analysis (Fig 4D-E).

**Figure 5:**
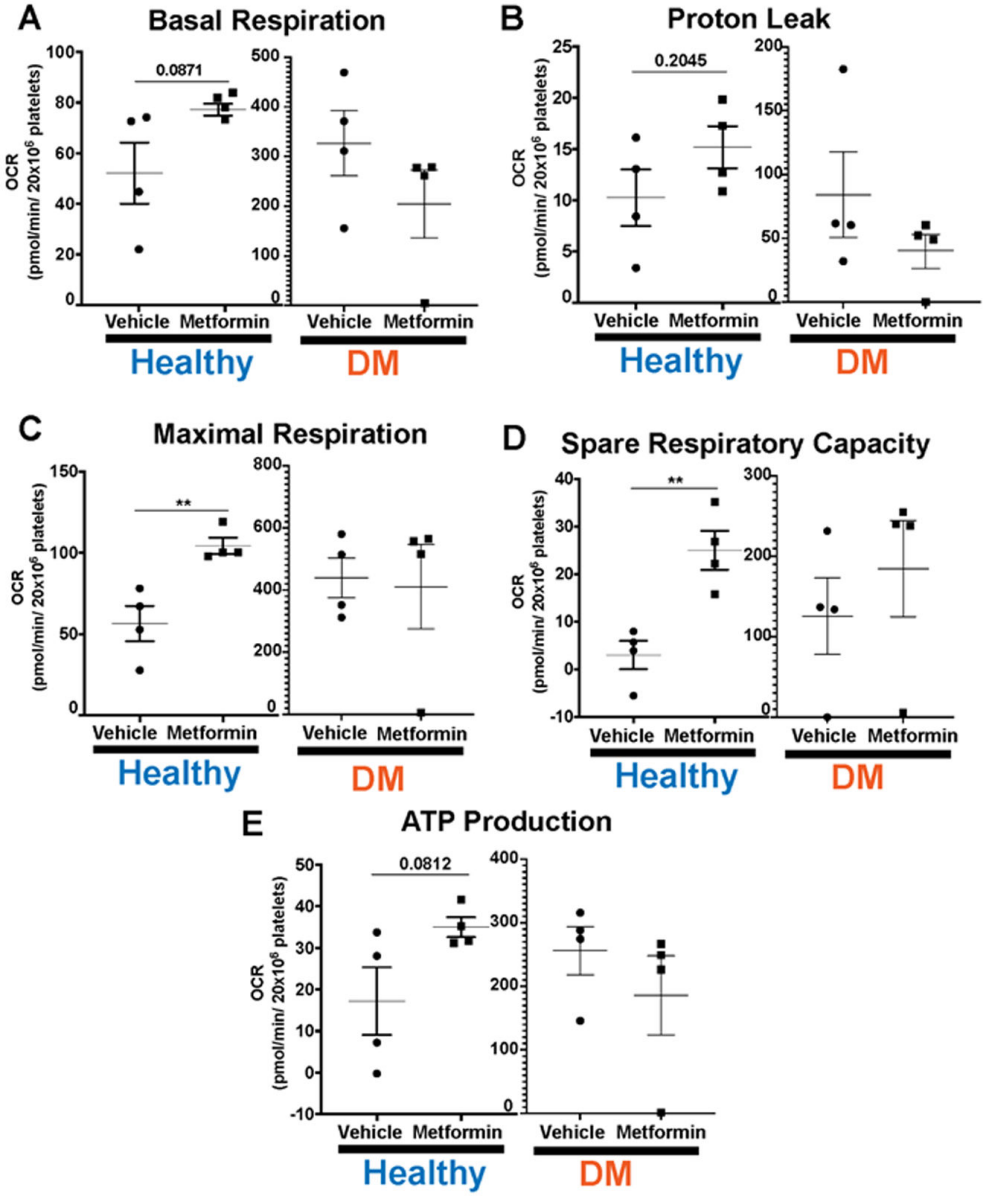
Platelet bioenergetic analysis of mitochondrial respiration after 7-Day Metformin Treatment DM mice have higher threshold of platelet mitochondrial bioenergetics compared to non-DM mice in all our platelet bioenergetic assays (A-E). Results show that DM mice do not evidence any statistically significant differences in platelet bioenergetic parameters between vehicle vs Metformin treated mice (A-E). Contrarily, non-DM (non-DM (healthy)) mice treated with Metformin show increase in maximal respiration and spare respiratory capacity (C-D). There is an increase in basal respiration, proton leak and ATP production in Metformin treated non-DM (healthy) mice but there were no significant statistical differences found (A-B, E). N of 3-4 subjects per group was used for Two-tailed t-test : p<0.01 (C-D).

Lastly, we evaluated platelet bioenergetics in both non-DM (healthy) and DM mice (Fig 5). DM mice show higher baseline platelet mitochondrial respiration [23], consistent with hyperglycemia-induced increase in glucose metabolism [24]. There were no differences with Metformin treatment in any of the parameters we interrogated in DM treated mice compared to controls (Fig 5). In contrast, high dose of Metformin increased maximal respiration and spare respiratory capacity in platelets from non-DM (healthy) mice compared to controls (Fig 5C-D). Metformin reflects an overall effect in non-DM (healthy) mice showing shorter time of occlusive thrombosis and shorter bleeding time that is not observed in DM mice.

## 4. Discussion

Our studies were aimed to better understand the effect of the commonly used high dose of Metformin treatment in diabetic arterial thrombosis. The novelty of our studies shows promising antiplatelet effects of commonly used preclinical dose of Metformin in arterial thrombosis. There have been multiple attempts to study such effects before, but no dose response studies have been carried out to distinguish Metformin for potential beneficial effects in disease [25, 26]. We have made striking observations on the distinct effects of Metformin in DM platelets. We found that Metformin decreases the susceptibility of DM mice to arterial thrombosis but not in non-DM (healthy) mice. Associated to these results, non-DM (healthy) mice treated with Metformin show shorter bleeding time with faster thrombotic occlusion. These are unexpected results that guided our related studies at platelet directed studies in our model. We expected DM mice to have increased basal platelet mitochondrial respiration, but we were surprised to find no evidence of an effect of Metformin in DM mice. Strikingly, Metformin increasing maximal respiration and spare respiratory capacity only in non-DM (healthy) mice.

Because of our initial findings described in Fig 2 and Fig 3 in non-DM (healthy) mice, we decided to further study the changes Metformin showed ex vivo by evaluating their platelet function uniquely focused in non-DM (healthy) mice. Therefore, we performed flow cytometry analysis of platelet activation and adhesion in DM mice, and we observed an increase in platelet activation (P-selectin) via Collagen stimulation with an opposite effect on GPIIb/IIIa with the same stimulant. Evidently, there was observed a shorter bleeding time in non-T2D mice when treated with Metformin though a greater amount of whole blood was bled. No difference was observed between tail bleed of DM mice treated with or without Metformin. This mirrors the results observed in the arterial thrombosis model. To determine whether metformin modulates platelet function in non-DM (healthy) mice, we assessed platelet aggregometry in non-DM (healthy) mice after 7-day treatment with Metformin vs vehicle treatment. There was a consistent increase in platelet aggregation and lag time in Metformin treated mice regardless of length of stimulation. We found the same increase in aggregation and lag time only in Metformin treated non-DM (healthy) mice. Platelet activation in non-DM (healthy) mice is driven predominantly by ADP stimulation, however ADP had a negligent effect in GPIIb/IIIa activation in non-DM (healthy) mice treated with Metformin. Since Metformin is an AMPKa activator, stimulation with ADP is fundamental for the therapeutic effect of Metformin in diabetic patients [10, 27].

Moreover, Metformin can replenish ATP levels by increasing ATP/ADP ratio particularly under hyperglycemic conditions [28]. Clinical studies have shown that there is a benefit in reducing platelet specific effects in a dose-dependent manner of Metformin but uniquely in T2D patients [7]. This correlates to our findings uniquely in DM mice, where there appears to be beneficial effects of Metformin that is not observed in healthy mice. Finally, we showed that these non-DM (healthy) mice had no development of kidney damage (Cystatin C) or associated changes in platelet activation using in vivo urinary thromboxane analysis. We believe that platelet activation results in Figure 2 correlate AMPKa dependence in DM. Lack of significant AMPKa pathway activation in non-DM (healthy) as occurs in diabetes might explain changes in aggregation. There is evidence of platelet activation by increase in P-selectin in almost all our groups. However, the important evidence to highlight is that Metformin increased platelet adhesion via GPIIb/IIIa activation under Collagen stimulation in non-DM (healthy) mice. Contrarily, in DM mice, under Collagen stimulation, platelet adhesion is decreased. This would explain why DM mice treated with Metformin have less arterial thrombus formation as it mimics a physiologic condition in vivo even though there is platelet activation with Collagen.

We need to point out the limitations of our studies. We will start by discussing the use a well-established model of hyperglycemia in DM [29, 30]. We allowed DM to develop for full 12 weeks before experimental studies. We started injections at 6 weeks of age and allowed an acclimation period after injections since the model is time- and STZ dose-dependent [18]. We compared STZ injected mice (DM) to non-STZ injected (non-DM (healthy)) mice. There have been other studies done previously in diabetic platelets and arterial thrombosis [9, 31]. However, we did find some differences in results compared to our studies. For example, Stolla et al in 2013 show results showing an effect in diabetic platelets in a model of FeCl3 arterial thrombosis. However, their results in the FeCl3 thrombosis model only present Mean Fluorescence Intensity quantification of Fibrinogen but not time of occlusion. Their mouse model was more indicative of a strong Type-1 Diabetes with less Insulin Resistance due to time (3 weeks vs 12 weeks) and the different dose of STZ used (50mg/kg vs 25mg/kg). We believe that the paradoxical effects we observe in non-T2D mice might be due to study is the high dose of Metformin which may have a particular off-targeted effect. Since these animals are mostly “healthy”, such high doses of Metformin have a distinctive paradoxical effect in “healthy” mice [32]. There is evidence that indicates high doses of Metformin causes alterations in coagulation factors, fibrinogen, and platelet homeostasis [33-35].

Components of the coagulation cascade and thrombin activity could be affected by such high dose of Metformin that counterbalance potential beneficial effects in healthy mice [16, 36]. However, those potential studies are beyond the scope of these studies but need to be further explored [33-35]. We decided to test this dose since promising effects had been observed in other small animal models of arterial thrombosis [9]. Another limitation is that we are using relatively young mice that are non-obese and as such the differences observed may be due to the altered effects of diet-induced obesity in other murine models of diabetes [37]. Importantly, we administered Metformin via oral gavage vs in drinking water which also increases the bioavailability and effect of Metformin [38]. These factors could potentially explain why we observe such differences in non-DM (healthy) mice [37]. We evidence almost no difference in time of occlusive thrombosis between non-DM (healthy) and DM mice. We believe that this is due to changes in lag time due to Metformin particularly in DM mice that are not evident in our 2-minute assay. Studies in other murine models of DM show that arterial thrombosis effects can be observed way beyond our established cutoff [39, 40].

Our conclusion is that the effect of Metformin might be difficult to mimic translationally due to the altered platelet responses in our in vivo mouse model that we are not being able to replicate exactly ex vivo [41]. However, there is benefit in reducing platelet specific effects in a dose-dependent manner of Metformin but apparently is unique to DM patients [7]. Therefore, we present data showing that Metformin decreases susceptibility to develop occlusive arterial thrombosis only in DM mice. We demonstrate an unexpected Metformin-induced increased propensity for thrombosis in non-DM (healthy) mice. Further studies will be needed to underpin the mechanisms by which dose-dependent effects of Metformin mediate ADP-regulated platelet function in DM.
